# Impact of Repeated Doses of Subcutaneous Esketamine on Acute Dissociative Symptoms in Treatment-Resistant Depression

**DOI:** 10.3390/ph16010031

**Published:** 2022-12-26

**Authors:** Lorena Catarina Del Sant, Luciana Maria Sarin, Ana Cecília Lucchese, Eduardo Jorge Muniz Magalhães, Marco Aurélio Tuena, Carolina Nakahira, José Alberto Del Porto, Acioly Luiz Tavares De Lacerda, Jair de Jesus Mari

**Affiliations:** 1 Department of Psychiatry, Federal University of São Paulo, São Paulo 04017-030, Brazil; 2 Affective Disorder Program, Federal University of São Paulo, São Paulo 04017-030, Brazil

**Keywords:** esketamine, treatment resistant depression, dissociation

## Abstract

Background: Esketamine has been approved by the US Food and Drug Administration (FDA) as an adjunctive treatment for use in conjunction with an oral antidepressant for patients with treatment-resistant depression (TRD), but dissociative symptoms are common adverse effects. Methods: A retrospective analysis of 394 subcutaneous esketamine injections given to 70 patients with TRD that were administered once a week during a six-week trial in conjunction with oral antidepressant therapy. Doses between 0.5 to 1.0 mg/kg were administered according to the patient’s response. Dissociative symptoms were assessed using the Clinician-Administered Dissociative States Scale (CADSS) 30 and 60 min after every weekly treatment (day 1, 8, 15, 22, 29 and 36). Results: Seventy patients received a total of 394 subcutaneous esketamine injections over six weeks. Over time, the evolution of CADSS scores demonstrated a significant mean difference of CADSS at 60 min post-injection (*p* = 0.010) throughout the six infusions. The mean CADSS scores at 60 min on day 22, 29 and 36 were similar. There were no differences between mean CADSS scores 30 min after the injections, no clinical correlation between response and dissociative symptoms, no correlation between time and demographic and clinical characteristics and no interactions between time and combined medication. Conclusions: Our results suggest that repeated subcutaneous esketamine doses are safe and well-tolerated regarding their acute dissociative and psychotomimetic symptoms. Symptoms usually peak at 30 min and decrease at 60 min post-injection, returning to their pretreatment levels at 120 min. Dissociative symptoms do not correlate with antidepressant response.

## 1. Introduction

Depression is the leading cause of disability worldwide [1]. Patients who do not achieve remission after two or more antidepressant therapies are diagnosed as having treatment-resistant depression (TRD) [2]. However, TRD is not a formal diagnosis, and there is no clear consensus on its definition. Moreover, it is not included in the DSM-5 or ICD-10. In this article, we used the definition used by the FDA for approval for esketamine treatment (a lack of clinically meaningful improvement in depressive symptoms after treatment with at least two different oral antidepressant medications as monotherapy, taken at adequate doses for adequate duration [at least 6 weeks] for their current episode of depression). Ketamine, which is a racemic mixture of R- and S-enantiomers, is an uncompetitive antagonist of the N-methyl-D-aspartate (NMDA) receptor. It has demonstrated a rapid and robust antidepressant effect [3]. Esketamine is the S-enantiomer of racemic ketamine and has a higher affinity for the NMDA receptor than the R-enantiomer. In March 2019, the United States Food and Drug Administration (FDA) approved the use of intranasal esketamine as an adjunctive treatment with oral antidepressants for patients with TRD and adults with major depression exhibiting acute suicidal ideation or behavior [4,5,6,7,8]. However, we were unable to use this particular administration route because it was not launched in Brazil until November 2020, which was after the data was collected. 

Both ketamine and its enantiomers can produce psychotic symptoms such as psychotomimetic symptoms, dissociation, perceptual disturbances, abnormal sensations, derealization and depersonalization [9]. In a clinical setting, symptoms are mild to moderate in severity, transient and limited to dosing days. Studies that use the intravenous route of administration tend to report more psychotomimetic or dissociative effects than those using other routes such as oral, subcutaneous or intramuscular administration [10]. Intravenous infusion is the most common method of administration of ketamine, although there is growing evidence that the subcutaneous route is preferable due to its low cost, safety, tolerability and effectiveness, and this route has already become established as choice in adult and geriatric patients with medical comorbidities [11,12,13,14,15].

This study aimed to investigate the impact of repeated subanesthetic doses of subcutaneous esketamine on acute dissociative symptoms in a retrospective naturalistic cohort of patients. 

## 2. Results

A total of 70 patients received 394 subcutaneous esketamine injections over six weeks, with 64 patients completing the treatment protocol. The patients received up to six injections over six weeks (mean injection count = 5.77; standard deviation = 0.7205), with a minimum of three injections. The mean dose of esketamine was 0.78 mg/kg. The esketamine doses were increased to 1.0 mg/kg in 50 (91.4%) patients due to their lack of response. Overall, 91.4% of patients had their doses increased at some time during the course of the treatment, and 34 (53.1%) patients were considered responders. The patients exhibited no dissociative symptoms before the procedure. The demographic and clinical characteristics of the sample are presented in Table 1. There were no serious adverse events due to psychotomimetic symptoms.

### 2.1. Dissociative Symptoms (CADSS Score) over Time

**Figure 1:**
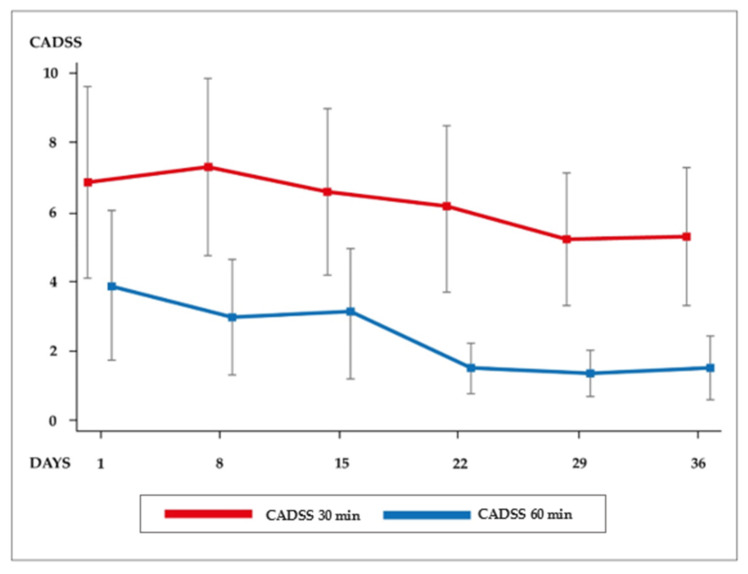
Mean CADSS scores 30 and 60 min after the infusions.

The internal consistency between the CADSS items was assessed via Cronbach’s alpha coefficient, and its value ranged from 0.710 (acceptable) to 0.945 (excellent). Over time, the evolution of 30 min CADSS scores demonstrated a significant mean difference compared with 60 min CADSS scores (*p* = 0.010) throughout the six infusions. Figure 1 shows that the CADSS scores for the first three infusions were similar and higher than those of the last three infusions. The mean CADSS scores following the fourth, fifth and sixth infusions at 60 min were similar (see Table 2). There were no significant differences between the CADSS scores over the six infusions at 30 min (*p* = 0.400). However, after adjustment by Bonferroni correction for multiple comparisons, the mean CADSS score at 30 min was higher than that at 60 min post-injection on days 1 (*p* = 0.040), 8 (*p* = 0.001), 15 (*p* = 0.011), 22 (*p* < 0.001), 29 (*p* = 0.003) and 36 (*p* = 0.004). After 120 min post-injection, all patients had no dissociative symptoms according to CADSS scores.

### 2.2. CADSS Scores over Time Adjusted by Dose

Flexible doses based on the patient’s response were used throughout the treatment (0.5 up to 1.0 mg/kg). The generalized estimating equations (GEE) model was adjusted with the CADSS score as the dependent variable, and the injections and doses over time were considered as predictor variables. Table 1 shows that dose was not significant for CADSS scores at both 30 min (*p* = 0.502) and 60 min (*p* = 0.055). However, a time effect that was not related to the dose was found but only for the CADSS score at 60 min (*p* = 0.006). Furthermore, the mean CADSS scores at day 8 and 15 did not differ from those at day 1, whereas the mean CADSS scores in subsequent injections were similar to each other and lower than those at the initial dose (four points less). Considering the dose for the mean CADSS score at 60 min (*p* = 0.055), a larger sample size could result in a dose effect on the CADSS score. 

### 2.3. Mean CADSS Score over Time and Treatment Response

The effects of the injections by group (responders vs. non-responders) were analyzed for time and group as well as for the interaction between group and time. No interactions were found between time and group for the mean CADSS scores at 30 min (*p* = 0.128) and 60 min (*p* = 0.588) (Table 2), indicating that the evolution of the mean CADSS scores was similar between groups. Therefore, treatment response was not clinically correlated with dissociative symptoms.

### 2.4. Mean CADSS Score over Time by Demographic and Clinical Characteristics

No interactions were found between time and demographic and clinical characteristics for the mean CADSS scores at 30 and 60 min (*p* = 0.971, *p* = 0.958, *p* = 0.685 and *p* = 0.077 at 30 min and *p* = 0.857, *p* = 0.975, *p* = 0.470 and *p* = 0.096 at 60 min for sex, age, diagnosis of unipolar and bipolar depression and anxiety, respectively), indicating that the evolution of the mean CADSS scores was similar between groups. Additionally, the mean CADSS scores at 30 min showed no effects of time (*p* = 0.654), sex (*p* = 0.155), age (*p* = 0.158), diagnosis of unipolar and bipolar depression (*p* = 0.444) or anxiety (*p* = 0.706). 

### 2.5. Mean CADSS Score over Time by Medication

No interactions were detected between time and combined medication (*p* = 0.406 and *p* = 0.890, respectively), drugs for anxiety (*p* = 0.577 and *p* = 0.470, respectively) or for psychosis (*p* = 0.717 and *p* = 0.639, respectively), suggesting that the evolution of the mean CADSS score was similar between different medications. Additionally, there were no time effects at 30 min and 60 min for all six injections (*p* = 0.872, *p* = 0.408 and *p* = 0.974 at 30 min and *p* = 0.557, *p* = 0.217 and *p* = 0.892 at 60 min for combined medication, drugs for anxiety and for psychosis, respectively) and at 30 min for medication (*p* = 0.567, *p* = 0.268 and *p* = 0.454 for combined medication, drugs for anxiety and for psychosis, respectively). However, a time effect was observed with drugs for anxiety (*p* = 0.019) at 60 min. Thus, for both groups with and without the use of drugs for anxiety, the mean CADSS scores at days 1 and 8 were similar and higher than those at days 22, 29 and 36. 

## 3. Discussion

This study revealed several findings related to the impact of repeated subcutaneous esketamine injections on acute dissociative symptoms in a real-world cohort of TRD patients with major depressive disorder or bipolar depression. Dissociative changes, assessed using the CADSS, occurred shortly after dosing, resolved 2 h post-dose and were limited to dosing days. Over time, CADSS scores demonstrated a significant mean difference at 60 min (*p* = 0.010) throughout the six infusions. These results were consistent with those of several other studies on ketamine and esketamine that were administered using different routes [5,6,8,16,17].

The mean CADSS score per dosing day was lower than those in previous ketamine intravenous studies [18]. The mean CADSS scores at days 1, 8 and 15 were higher than those at days 22, 29 and 36, indicating possible dissociative tolerance over time after 60 min. The intranasal esketamine route is also associated with dissociative effects, which generally attenuate during the treatment [4,5,6,19]. Popova et al. (2019) [8] demonstrated an attenuation in CADSS scores with IN esketamine doses of 56 and 84 mg over time.

After analyzing the CADSS scores adjusted by dose, different doses were not significant in terms of CADSS scores at both 30 min and 60 min. However, the *p*-value of 0.055 for the dose at 60 min suggested that dose increases could have affected the CADSS score at 60 min if more patients had been included in this study. A previous study [20] showed that both 0.5 mg/kg and 1 mg/kg doses led to significantly greater CADSS scores at 40 min after ketamine infusion than after the active placebo, although there were no statistically significant correlations between the changes in CADSS scores at 40 min after infusion. Another study [14] demonstrated a subcutaneous ketamine dose-dependent increase in CADSS scores at 30 min that returned towards the baseline by 60 min, while a mixed model analysis identified a significant dose × time interaction. The IN route [8] did not find a dose correlation with CADSS scores at 56 and 84 mg. Therefore, whether the dissociative effects of ketamine are dose-dependent or not remains unclear.

Racemic ketamine and esketamine have a well-established association with dissociation adverse effects, which are broadly defined as altered consciousness and awareness of self, the environment and reality. Pharmacological effects usually peak within 1 h after ketamine administration and resolve by 2 h post-injection [10]. 

Esketamine has been investigated in a number of double-blind, randomized studies of TRD, which mostly used bi-weekly intranasal (IN) administration [5,6,8,17]. However, differences among the study designs limit direct comparisons. The IV route was also investigated in two single-dose studies [16,17]. Singh et al. (2016) [17] reported dissociative symptoms, such as visual hallucinations, depersonalization, derealization and disturbances in logical thinking.

Recent studies have suggested that the CADSS has limitations as a tool for measuring the acute effects of ketamine administration [18,21]. The authors of these studies hypothesized that since the scale was designed to assess dissociative symptoms in patients with PTSD, it might not truly capture ketamine’s dissociative symptoms. This supports the view that part of the CADSS scale assesses phenomenology that is not prominent in the acute ketamine experience. 

Dissociative symptoms have been shown to be a predictor of antidepressant responses [22,23]; however, this relationship remains unclear. Acevedo-Diaz et al. (2020) [24] showed that dissociative effects did not mediate antidepressant responses to ketamine. A study that used IN administration of esketamine [8] reported that the proportion of responders was similar in those with and without dissociative symptoms. Our findings are consistent with those studies that did not find any correlation between dissociative symptoms and antidepressant responses. 

Our results demonstrated no statistical differences between dissociative symptoms and demographic and clinical characteristics, including age, sex, diagnosis (unipolar or bipolar), anxiety comorbidity and different medications (drugs for anxiety and/or for psychosis). Derntl et al. (2019) [25] reported different results and demonstrated that depersonalization and amnestic symptoms in the ketamine group were significantly higher in men than in women. Moreover, older men experienced fewer dissociative symptoms, suggesting a sex-specific protective effect of older age. These discrepancies in results might be attributed to the inclusion criteria, as the study by Derntl et al. (2019) [25] was focused on healthy young adults (18–30 years).

Previous studies have shown that subcutaneous esketamine is safe, advantageous and effective when combined with oral antidepressant therapies even in patients with clinical comorbidities and in older adults [11,13,14,15]. Subcutaneous esketamine administration is more economical than IV or IN routes since there is no need for hospitalization or infusion pumps. This study has clinical significance because it demonstrates that subcutaneously administered esketamine is well tolerated and may be a feasible treatment option for TRD due to its efficacy, few side effects, low cost (estimated cost of USD 3 per dose) and low complexity. This suggests that this treatment is suitable for adoption in the public health system because it is the most cost-effective treatment.

The current study has several limitations which should be considered. Our sample was heterogeneous since only patients with clinical and psychiatric comorbidities were included, and patients maintained their usual oral psychotropic medication. There was a lack of randomization and a control group. Furthermore, it was a flexible-dose study, where an ascending dose was employed depending on the treatment response rather than a randomized or fixed-dose design. Moreover, our patients were referred from an academic program and had a prior history of multiple antidepressant therapies, making the sample less representative of severe TRD patients.

## 4. Materials and Methods

This study was a retrospective analysis assessing the impact of low-dose subcutaneous esketamine (Ketamin NP, Cristália Prod. Quím. Farm. Ltda., Itapira, Brazil) on dissociative adverse events, and it comprised a large case series in which 394 subcutaneous esketamine injections were administered to 70 TRD outpatients. All patients were referred to the esketamine program of the Department of Psychiatry at the Universidade Federal de São Paulo (UNIFESP) between April 2017 and December 2018. The patients were 15 to 66 years of age, had a moderate to severe current depressive episode, were either unipolar or bipolar and had scores > 20 on the Montgomery–Åsberg Depression Rating Scale (MADRS). All patients had a prior history of non-response to at least two antidepressants or mood stabilizers for at least six weeks at an effective dose in the current episode. Complete details of sample selection were reported elsewhere [11,26]. The study was approved by the UNIFESP Ethical Committee (no. 434/2018). 

### 4.1. Procedures

Esketamine was administered as subcutaneous bolus injections in the abdominal region. One injection per week was administered over a six-week period in conjunction with oral antidepressant therapy. This was a multiple-dose treatment course with, when necessary, an ascending dose protocol (with an initial dose of esketamine of 0.5 mg/kg). Higher doses of esketamine of 0.75 up to a maximum of 1 mg/kg were subsequently administered according to the patient’s response, assessed seven days post-dose, where response was defined as a ≥50% decrease in the patient’s total MADRS baseline score. If a response was not achieved (<50% decrease in the patient’s total MADRS baseline score), the patient’s esketamine dose was increased first to 75 mg/kg and then, if necessary, to a maximum of 1.0 mg/kg. On the day of each dose (days 1, 8, 15, 22, 29 and 36) dissociative symptoms were assessed using the Clinician-Administered Dissociative States Scale (CADSS) 30 and 60 min post-injection [27].

### 4.2. Statistical Analysis

The CADSS scores over the six infusions were our primary outcome of interest. Initially, the data was analyzed descriptively. Absolute and relative frequencies were presented for categorical variables, while summary measures were used for numerical variables. The internal consistency between the CADSS items was assessed using Cronbach’s alpha coefficient. The Last Observation Carried Forward approach, also known as end-point analysis, was used to impute any missing data. To analyze the dissociative symptom scores, a Generalized Estimating Equations (GEE) model was used with an identity link function and normal marginal distribution. The GEE approach allows the incorporation of the dependent variables of different distributions between the observations of the same patient resulting from the repeated measures carried out over time. For all statistical tests, a significance level of 5% was adopted. The GEE models were estimated using STATA 12. For other analyses, the statistical software SPSS 20.0 (IBM Corp. Released 2011. IBM SPSS Statistics for Windows, Version 20.0., Armonk, NY, USA) was used.

## 5. Conclusions

Our results suggest that repeated subcutaneous esketamine doses are well tolerated in terms of acute dissociative symptoms. Symptoms usually peak at 30 min, decrease at 60 min post-injection and return to their pre-treatment levels at 120 min. Dissociative symptoms do not correlate with antidepressant responses and may attenuate over time. Further research and clinical trials are essential for assessing the long-term dissociative effects of subanesthetic doses of subcutaneous esketamine.

## Figures and Tables

**Table 1:** Demographic and clinical characteristics of the sample.

Demographic Variables		Clinical Variables		Current Pharmacotherapies	
Variables	N/Mean (%/SD)	Variables	N/Mean (%/SD)	Variables	N (%)
Age (years)	40.31 (12.67)	**Diagnosis**		Adjunctive Treatment	64 (91.42)
Gender (female)	45 (64.30)	MDD BD	39 (55.71) 31 (44.29)	Antidepressants	48 (68.57)
Education (college graduate)	41 (55.57)	**Severity**		Antipsychotics	41 (58.57)
Occupational status (employed)	16 (22.86)	Baseline MADRS	33.6 (6.32)	Lithium	30 (42.85)
		Maudslay *	11.09 (2.03)	Anticonvulsants	37 (52.85)
		Anxiety Disorder	31 (44.29)	Benzodiazepines	26 (37.14)
		BMI	29.1 (7.5)		
		Clinical Comorbidity	57 (81.42)		
		Obesity	24 (34.29)		
		Hypertension	12 (17.14)		

* MSM the Maudslay Staging Method.

**Table 2:** CADSS mean score differences over time adjusted by dose (N = 70).

	30 min		60 min	
	Coefficient (CI 95%)	*p*	Coefficient (CI 95%)	*p*
Day 1		0.483		0.006
Day 8	0.01 (−2.56–2.58)	0.994	−1.62 (−3.46–0.22)	0.084
Day 15	−1.08 (−4.28–2.11)	0.507	−2.21 (−4.45–0.03)	0.053
Day 22	−1.62 (−5.03–1.80)	0.354	−4.01 (−6.39–−1.63)	0.001 ^a^
Day 29	−2.59 (−6.08–0.89)	0.145	−4.26 (−6.69–−1.84)	0.001 ^a^
Day 36	−2.52 (−6.06–1.02)	0.164	−4.10 (−6.56–−1.63)	0.001 ^a^
Dose	2.29 (−4.40–8.98)	0.502	4.38 (−0.10–8.86)	0.055
Constant	5.74 (1.63–9.85)	0.006	1.65 (−1.04–4.34)	0.230

^a^ Time effect not related to dose.

## Data Availability

Data is available within article.
